# Characterization and phylogenetic relationship of the complete chloroplast genome of a Chinese traditional medicinal plant *Potentilla anserina* L

**DOI:** 10.1080/23802359.2022.2119817

**Published:** 2022-09-15

**Authors:** Chuyu Tang, Xiuzhang Li, Jianbo Chen, Jing Liang, Tao Wang, Yuling Li

**Affiliations:** State Key Laboratory of Plateau Ecology and Agriculture, Qinghai Academy of Animal and Veterinary Science, Qinghai University, Xining, China

**Keywords:** *Potentilla anserina*, chloroplast genome, phylogenetic analysis

## Abstract

*Potentilla anserina* L. is an important traditional Chinese medicinal herb and edible plant with a long usage history. As an indispensable sustainable resource, it has various pharmacological functions and active ingredients. Here, we report its complete chloroplast (cp) genome for the first time. The complete chloroplast genome of *Potentilla anserina* L. (OL678458) was 155,659 bp in length and contained a pair of inverted repeat regions (IRa and IRb, 25,947 bp), a large single-copy region (LSC, 85,052 bp), and a small single-copy region (SSC, 18,713 bp). A total of 118 functional genes were observed in this cp genome, including 80 protein-coding genes, 30 transfer RNA genes, and eight ribosomal RNA genes. Phylogenetic analysis indicated that *P. anserina* has the closest relationship with *Potentilla lineata*.

The medicinal plant *Potentilla anserina* (Linnaeus 1753) belongs to the *Potentilla* genus of Rosaceae and is widely distributed in the temperate, frigid alpine zones of the Northern Hemisphere (Wang et al. 2016; Xia and You 2011). In China, it is commonly distributed in the western areas, particularly in the Qinghai-Tibetan Plateau (Shi et al. 2021). The root of *P. anserina*, known as 'juémá,’ is found in traditional Chinese medicine (TCM) studies containing polysaccharides, steroids, triterpenoids, flavonoids, phenolic acids, coumarins, etc. (Kovaleva and Abdulkafarova 2011). It has been reported that *P. anserina* can function as an antioxidant, immunomodulatory, antifibrinolytic, antitussive, and expectorant agent (Chen et al. 2010; Kovaleva and Abdulkafarova 2011; Tao et al. 2015). Therefore, further characterization and analysis of this plant is of great social value for research and utilization.

In the era of molecular biology and genome analysis, the chloroplast genome provides indispensable genetic information for plant classification, phylogeny, and species identification (Zhang et al. 2021), and it will directly mirror the variation of the plant in the process of evolution and indicate the origin of species and migration (Wei et al. 2021). However, there is no research on the chloroplast genome of *P. anserina* currently. To fill the gap in population genetics research and to facilitate its utilization, we report the first complete *P. anserina* chloroplast genome in this study.

Fresh *P. anserina* leaves were collected from a plant in Qilian County, Haibei Tibetan Autonomous Prefecture of Qinghai Province, China (E100°14′07″, N37°59′49″), in August, 2021. Voucher specimens were deposited at the Qinghai Academy of Animal Husbandry and Veterinary Sciences, Qinghai University (voucher No. QH0241, Xiuzhang Li, xiuzhang@qhu.edc.cn).

Genomic DNA was extracted from the leaves using the modified CTBA method (Doyle 1987). A 350 bp library of DNA samples was constructed using the whole-genome shotgun method, and the total *P. anserina* genome was sequenced with 150 bp pair-end mode on the Illumina NovaSeq sequencer. Qualified clean reads were assembled using NOVOPlasty (Dierckxsens et al. 2017) with *Potentilla freyniana* (NC_041210.1) as the initial reference. Finally, the total *P. anserina* genome sequence was annotated using GeSeq (Michael et al. 2017), and a new annotated chloroplast genome sequence was submitted to GenBank (OL678458). Phylogenetic trees were generated using maximum likelihood (ML) analysis.

The chloroplast genome of *P. anserina* (OL678458) was 155,659 bp in length (GC ratio was 36.78%) and had four subregions: 85,052 bp of large single copy (LSC; 34.59%) and 18,713 bp of small single copy (SSC 30.55%) regions separated by 25,947 bp of inverted repeat (IR; 42.6%). The overall A + T content of the cp genome was 63.22%, whereas the corresponding values of the LSC, SSC, and IR regions were 65.41, 69.45, and 57.4%, respectively. There were 118 functional genes observed in the cp genome of this herb, including 80 protein-coding genes, 30 tRNA genes, and 8 ribosomal RNA genes. In the circular chloroplast genome of *P. anserina*, most genes appeared in a single copy, whereas 15 genes occurred as two copies, including four protein-coding (*rpl2*, *rpl7, rpl23*, and *rpl12*), four rRNA (*rrn4.5, rrn5*, *rrn16*, and *rrn23*), and seven tRNA (*trnl-CAU*, *trnL-CAA*, *trnV-GAC*, *trnl-GAU*, *trnA-UGC*, *trnR-ACG,* and *trnN-GUU*) genes were duplicated in the IR regions.

Based on the complete chloroplast genomes of 17 species within *Potentilla* and other species, we used the maximum likelihood (ML) method of IQ-TREE to construct a phylogenetic tree to analyze close relationships (Nguyen et al. 2015). *Haloxylon persicum* (NC_027669.1) and *Salsola abrotanoides* (NC_057096.1) were considered outgroups and 31 species were clustered into two branches by ML analysis. The phylogenetic results showed that *P. anserina* was most related to *Potentilla lineata* with strong bootstrap values (Figure 1), belonging to the *Potentilla* genus of Rosaceae. This study provides a theoretical basis for future protection and research of this species, which will help us further understand the developmental course of organisms.

**Figure 1.**
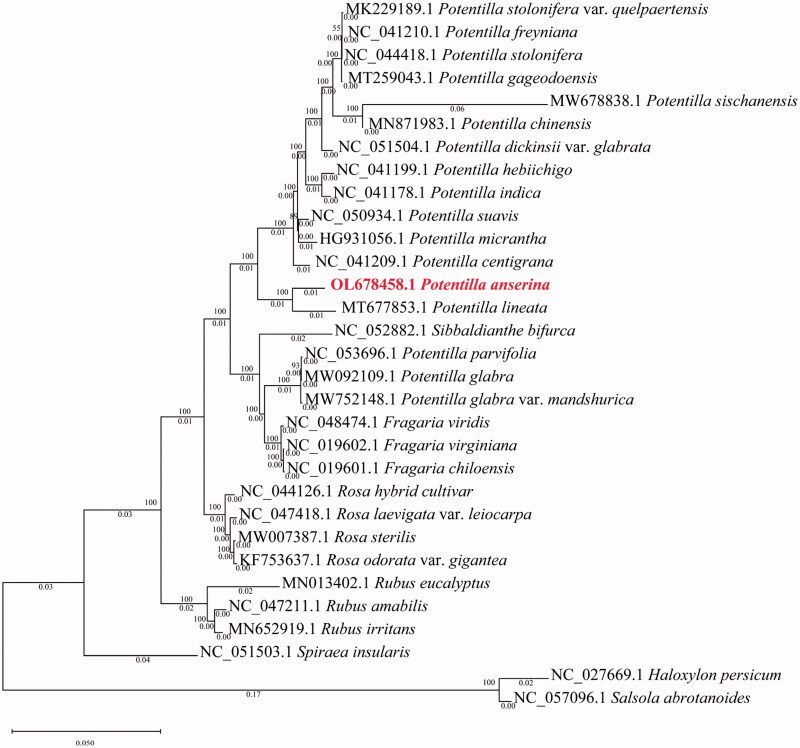
The maximum-likelihood phylogenomic tree is based on 31 complete chloroplast genome sequences. The *Potentilla anserina* is marked in red and bootstrap values are listed for each branch.

## Ethical approval

The field studies about collecting *Potentilla anserina* samples complied with the Grassland Law of the People’s Republic of China and obtained the permission of the Qinghai Academy of Animal and Veterinary Science.

## Author contributions

Chuyu Tang was involved in the conception and drafted the manuscript. Xiuzhang Li analyzed the interpretation of the data-designed experiments and revised them critically for intellectual content. Jianbo Chen, Jing Liang, and Tao Wang took field samples. Yuling Li provided reagents and experimental equipment. All authors reviewed the manuscript and agreed to be accountable for all aspects of the work.

## Data Availability

The genome sequence data that support the findings of this study are openly available in GenBank of NCBI at (https://www.ncbi.nlm.nih.gov/) under the accession No. OL678458 (https://www.ncbi.nlm.nih.gov/nuccore/OL678458). The associated BioProject, SRA, and Bio-Sample numbers are PRJNA796783 (https://www.ncbi.nlm.nih.gov/bioproject/PRJNA796783/), SRA: SRR17611292 (https://www.ncbi.nlm.nih.gov/sra/SRR17611292/), and SAMN24917766 (https://www.ncbi.nlm.nih.gov/biosample/?term=SAMN24917766) respectively.
